# Navigating the Pitfalls of Lung Cancer Screening: A Case Study on the Risks and Costs of Negative Screenings

**DOI:** 10.7759/cureus.59844

**Published:** 2024-05-07

**Authors:** Hoda Shabpiray, Jerrin George, Shivani Patel, Mani Khorsand Askari

**Affiliations:** 1 Department of Medicine, The University of Toledo College of Medicine and Life Sciences, Toledo, USA

**Keywords:** healthcare costs, diagnostic delay, low-dose computed tomography, false-negative results, lung cancer screening

## Abstract

Lung cancer is the leading cause of cancer-related deaths in the United States. Low-dose computed tomography is the preferred screening method for high-risk individuals. However, with a false-negative rate reaching 15%, this method can underestimate disease prevalence and delay necessary treatment. This case examines a 61-year-old female smoker with chronic obstructive pulmonary disease who initially received a negative result from screening. Her imaging findings were categorized as Lung Imaging Reporting and Data System (Lung-RADS) 2 but advanced to small cell lung carcinoma. This progression emphasizes the imperative of thoroughly evaluating screening results and patient history. False-negative results from screenings have profound implications, leading to delayed diagnoses, adversely affecting patient outcomes, and increasing healthcare costs. The necessity for vigilant follow-up enhanced diagnostic precision and transparent communication about limitations is paramount. An economic analysis emphasizes the significant financial impact of diagnosing lung cancer at advanced stages, highlighting the need for timely and accurate diagnostics. Comprehensive strategies, such as physician education, patient awareness, and stringent quality control, are crucial to improving the efficacy of lung cancer screening. Addressing the issue of false negatives is vital for enhancing early detection rates, decreasing healthcare expenses, and advancing patient care in lung cancer management. Continuous evaluation and adjustment of screening protocols are essential to reduce risks and optimize outcomes.

## Introduction

Lung cancer is the leading cause of cancer deaths in the United States [1]. The United States Preventive Services Task Force recommends annual screening for all adults between the ages of 50 and 80 years old with a 20-pack-year smoking history or more who are current smokers or have quit smoking within the past 15 years [2]. The reported false-negative rate for lung cancer screenings can reach as high as 15% [3]. This statistic comes from examining screening results, in which some cases of lung cancer were initially missed but subsequently detected, indicating that the initial screenings might underestimate the actual incidence of the disease. The accuracy of these results can be affected by several elements, such as the radiological assessment of the scans, the procedures for handling observed nodules, and the judgments made during multidisciplinary meetings. Furthermore, a screening interval of more than two years may also increase the likelihood of false negatives [3,4]. We present a patient with a negative lung cancer screening who was diagnosed with advanced lung cancer seven months after negative screening results. 

The abstract of this article was previously presented as a meeting abstract at Dr. Lance Dworkin Department of Medicine Annual Research Symposium at The University of Toledo College of Medicine and Life Sciences Meeting on September 28, 2023. 

## Case presentation

**Figure 1:**
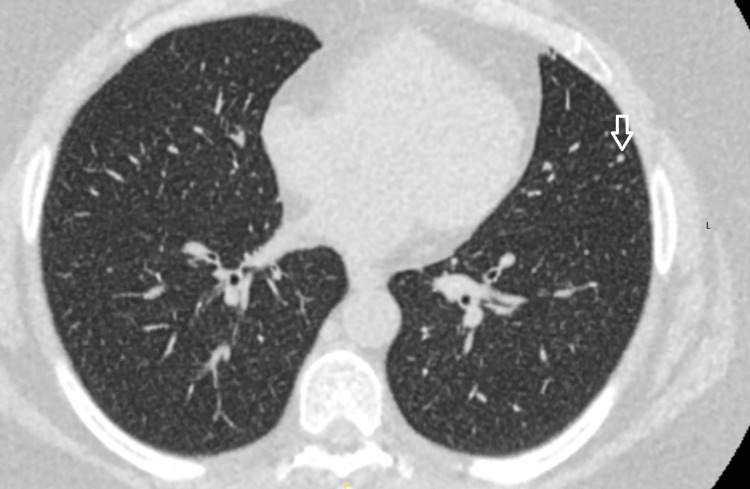
Low-dose computed tomography imaging showing a 3-mm nodule on the left side

A 61-year-old female with a significant medical history of chronic obstructive pulmonary disease (COPD) and a substantial 40-pack-year smoking history underwent a screening scan that initially indicated no malignant findings (Figure 1). Her screening results, classified under the Lung Imaging Reporting and Data System (Lung-RADS) as category 2, suggested benign nodules. The radiologist reported the findings as "Calcified granulomas noted. Scattered regions of centrilobular nodularity involving both lungs. Subtle diffuse centrilobular nodularity suggests the possibility of respiratory bronchiolitis or other airway process. There is a 3 mm lingular lung nodule that is not definitively calcified. This is unchanged from the prior exam. Category 2 - Benign screen. Continue annual screening in 12 months." The annual follow-up scan was recommended based on the recommendations of the American College of Radiology [5]. 

**Figure 2:**
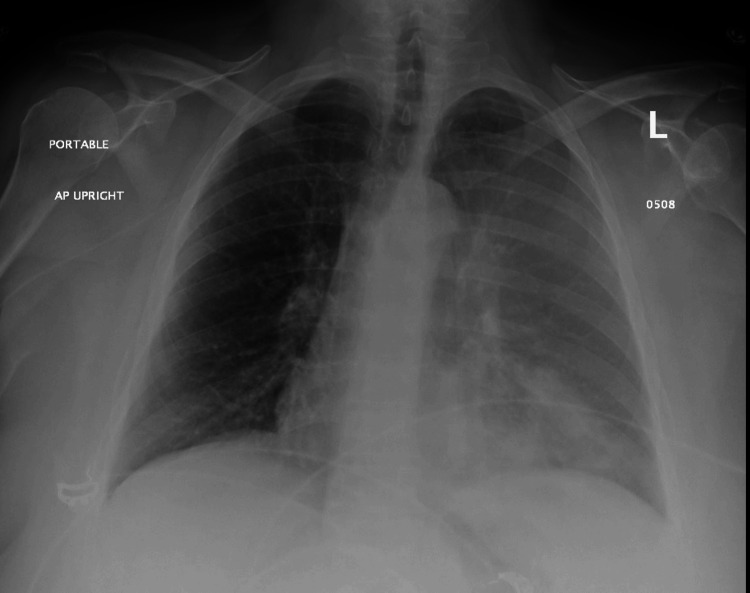
Chest X-ray at the time of presentation with pulmonary symptoms showing patchy infiltrates

**Figure 3:**
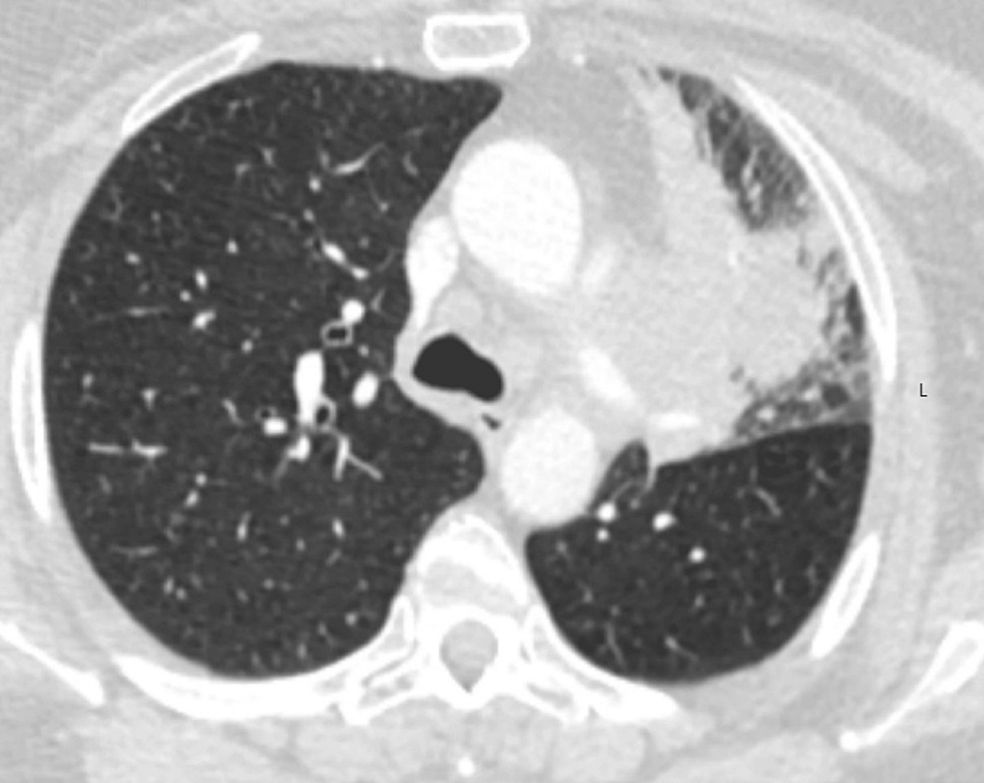
Chest computed tomography scan showing the mass on the left side

However, within seven months post-screening, the patient developed clinical symptoms, including shortness of breath and a productive cough, leading to her hospital presentation. Imaging studies at this time, specifically a chest X-ray, revealed patchy infiltrates, prompting treatment for presumed left upper lobe pneumonia and COPD exacerbation with a regimen of Augmentin, azithromycin, and prednisone (Figure 2). Persisting and exacerbating symptoms led to further investigations 10 days later, where a chest computed tomography scan identified a suspicious mass in the left upper lobe (Figure 3).

A lung biopsy confirmed the presence of small cell carcinoma. Further diagnostic work, including bone scans, revealed osseous metastasis, categorizing cancer as an extensive stage according to the Veterans' Administration Lung Study Group staging system [6]. The patient commenced treatment with carboplatin/etoposide but faced escalating adverse effects, leading to discontinuation after four cycles. Subsequent imaging demonstrated significant skeletal disease progression. With increasing spinal pain, she was advised for palliative radiotherapy, which she completed, receiving the last session four months following her diagnosis. Unfortunately, the patient expired five months after the diagnosis. 

## Discussion

Annual screenings for lung cancer have significantly reduced mortality by facilitating early detection. Over a 15-year period, the lung cancer screening intervention totaled $27.8 billion in costs and resulted in 985,284 quality-adjusted life years (QALYs) gained, leading to a cost-utility ratio of $28,240 per QALY gained [7]. Despite this success, the process is marred by a considerable incidence of false positives and false negatives. While extensive research has delved into the implications of false positives, the impact of false negatives, which can be as high as 15%, is equally concerning. Such false-negative results in lung cancer screenings can lead to delayed diagnoses and treatments, profoundly affecting the health outcomes of the screened population. Therefore, understanding and mitigating these errors is crucial for improving the efficacy and reliability of lung cancer screenings [8,9]. 

In the case under discussion, a patient exhibited symptoms indicative of lung carcinoma diagnosed seven months following a negative screening. This screening result can lead to a misplaced sense of security, causing a delay in seeking further medical attention when symptoms have started. Given her extensive history of smoking, the patient might have been unduly comforted by the negative lung cancer screening result, leading the patient and the providers to attribute serious symptoms like shortness of breath and coughing to less severe conditions rather than the possibility of lung cancer. This scenario underscores the critical need for vigilant follow-up and consideration of patient history and symptoms, even when initial screening results are negative. 

The false sense of security stemming from a negative screening result may not only affect the patient but can also permeate among healthcare providers, potentially leading to procrastinated diagnosis, treatment, and, consequently, suboptimal outcomes. Upon assessing the comprehension of screening processes among physicians, it emerged that most primary care doctors possessed an overinflated trust in the effectiveness of screenings. This confidence often lacked grounding in empirical evidence. Clinicians must emphasize to patients that, although their screening results are benign and suggest a very low lung cancer probability, this classification does not eliminate the future risk of developing lung cancer. Continual monitoring and regular follow-up screenings remain essential. This overconfidence can hinder the critical analysis of screening results and the necessary vigilance in patient follow-up, underscoring the importance of grounding medical decisions in evidence-based practices to enhance patient care outcomes [10]. 

It's crucial for physicians not only to have a thorough understanding of screening tools but also to communicate their limitations, including the relatively high false-negative rates, to their patients. A lack of comprehensive knowledge regarding these screening tools may affect the clinical judgment of some physicians, potentially explaining why lung cancer was not considered in the initial differential diagnosis when the patient presented with symptoms.

In this context, it's imperative to also consider lung adenocarcinoma in non-smokers, which is significantly influenced by genetic and epigenetic alterations, unlike its smoking-related counterparts. Studies highlight mutations in genes such as EGFR, ALK, and ROS1 predominantly in non-smokers, suggesting a distinct pathogenesis [11]. Moreover, epigenetic modifications like DNA methylation and histone alterations, often due to environmental factors other than tobacco smoke, play a crucial role in tumor development and progression [12]. Understanding these factors is vital for targeted therapeutic strategies and underscores the need for comprehensive patient evaluation irrespective of smoking status. 

In the National Lung Screening Trial, interval lung cancer among patients with negative screening results was uncommon. It is important to highlight that the occurrence of cancer at an advanced stage and high mortality rate shortly after a negative screening implies that these tumors were likely more aggressive or of a higher grade than those detected during screening. It is postulated that their outcomes might not have benefited from an earlier diagnosis [13]. Studies on non-small cell lung cancer have shown that delays in initiating surgical treatment beyond 12 weeks from diagnosis are correlated with increased recurrence rates and decreased survival rates, highlighting the critical need for timely and accurate diagnostic processes [13]. However, it is worth noting that screening focuses on detecting non-small cell lung cancer early instead of small cell lung cancer, as the latter is less prevalent and often spreads too rapidly to be consistently identified at an early, treatable stage through screening [4].

The economic toll of cancer is significant, placing a substantial burden on both patients and healthcare infrastructures. In 2015 alone, cancer's medical care costs soared to a staggering $183 billion, underlining the disease's financial strain. This indicates the vast resources devoted to diagnosis, treatment, and continuous care, highlighting the critical need for efficient strategies to reduce these economic impacts [14]. 

The economic consequences of delayed cancer diagnosis, mainly due to false-negative screenings, are severe for patients. Research tracking the costs for individuals with various cancers, such as lung, prostate, ovarian, colorectal, cervical, and breast, over a period extending to four years post-diagnosis showed that costs increase sharply with advanced-stage diagnosis. In particular, lung cancer patients diagnosed at stage 4 faced average annual costs of $418,591 in the first year post-diagnosis, in stark contrast to $161,116 for those diagnosed at stage 1, vividly demonstrating the financial implications of early versus late cancer detection and emphasizing the economic benefits of timely diagnosis and the high costs associated with diagnostic delays [14].

Additionally, hospital systems may face escalating costs when considering the potential malpractice-related expenses. An analysis of malpractice claims in 253 breast cancer cases revealed that a delayed diagnosis was the primary complaint in 82% of these cases, underscoring the prevalence of this issue in legal disputes. The financial consequences are significant, with the average settlement or award amount for plaintiffs reaching $978,858. This figure reflects the direct costs of malpractice claims and highlights the broader financial and reputational risks that healthcare providers face when diagnostic errors occur. Thus, improving diagnostic accuracy and reducing the time to diagnosis is critical for patient outcomes, mitigating financial liabilities, and enhancing the overall stability of healthcare systems [15]. 

## Conclusions

Lung cancer screening via low-dose computed tomography involves a shared decision-making process where patients are informed about the benefits and risks, including the potential for false-positive results and subsequent evaluations. However, it is also crucial to educate patients about the limitations of low-dose computed tomography, including the risks of false negatives and the possibility of lung cancer being diagnosed between annual screenings. To optimize lung cancer screenings, it is imperative to tackle the issue of false negatives, which can hinder timely diagnosis and negatively impact patient health. The highlighted case emphasizes the need for diligent follow-up and clear communication about screening limitations. Enhancing diagnostic precision, thorough physician education, and comprehensive patient information are crucial for reducing false-negative risks. This approach promotes early cancer detection, decreases healthcare expenditures, and elevates patient care quality, thus bolstering the efficacy and dependability of lung cancer screening initiatives. Effective lung cancer screening requires accurate decision-making, patient education on the potential for false results, and the necessity of follow-up assessments. False negatives often arise from under-recognized or underestimated nodules, typically classified in lower-risk categories. Regular quality control and review of classifications are essential for reducing false negatives and improving diagnostic accuracy, thereby preventing delayed treatment and perpetuating risky health behaviors that increase patient risks and healthcare costs. 

## References

[1] (2024). Key statistics for lung cancer. https://www.cancer.org/cancer/types/lung-cancer/about/key-statistics.html.

[2] (2024). Lung cancer: screening. https://www.uspreventiveservicestaskforce.org/uspstf/recommendation/lung-cancer-screening.

[3] Mascalchi M, Picozzi G, Puliti D (2023). Lung cancer screening with low-dose CT: what we have learned in two decades of ITALUNG and what is yet to be addressed. Diagnostics (Basel).

[4] (2024). United States Preventive Services Taskforce: lung cancer: screening. https://www.uspreventiveservicestaskforce.org/uspstf/recommendation/lung-cancer-screening.

[5] (2024). Lung CT Screening Reporting & Data System (Lung-RADS®). https://www.acr.org/Clinical-Resources/Reporting-and-Data-Systems/Lung-Rads.

[6] Kalemkerian GP (2012). Staging and imaging of small cell lung cancer. Cancer Imaging.

[7] Villanti AC, Jiang Y, Abrams DB, Pyenson BS (2013). A cost-utility analysis of lung cancer screening and the additional benefits of incorporating smoking cessation interventions. PLoS One.

[8] Aberle DR, Adams AM, Berg CD (2011). Reduced lung-cancer mortality with low-dose computed tomographic screening. N Engl J Med.

[9] Bartlett EC, Silva M, Callister ME, Devaraj A (2021). False-negative results in lung cancer screening-evidence and controversies. J Thorac Oncol.

[10] Wegwarth O, Schwartz LM, Woloshin S, Gaissmaier W, Gigerenzer G (2012). Do physicians understand cancer screening statistics? A national survey of primary care physicians in the United States. Ann Intern Med.

[11] Smolle E, Pichler M (2019). Non-smoking-associated lung cancer: a distinct entity in terms of tumor biology, patient characteristics and impact of hereditary cancer predisposition. Cancers (Basel).

[12] Brzeziańska E, Dutkowska A, Antczak A (2013). The significance of epigenetic alterations in lung carcinogenesis. Mol Biol Rep.

[13] Gierada DS, Pinsky PF, Duan F (2017). Interval lung cancer after a negative CT screening examination: CT findings and outcomes in National Lung Screening Trial participants. Eur Radiol.

[14] McGarvey N, Gitlin M, Fadli E, Chung KC (2022). Increased healthcare costs by later stage cancer diagnosis. BMC Health Serv Res.

[15] Lee MV, Konstantinoff K, Gegios A, Miles K, Appleton C, Hui D (2020). Breast cancer malpractice litigation: a 10-year analysis and update in trends. Clin Imaging.

